# NK Cell-Based Glioblastoma Immunotherapy

**DOI:** 10.3390/cancers10120522

**Published:** 2018-12-18

**Authors:** Irene Golán, Laura Rodríguez de la Fuente, Jose A. Costoya

**Affiliations:** Molecular Oncology Laboratory MOL, Departamento de Fisioloxia, CiMUS, Facultade de Medicina, Universidade de Santiago de Compostela, IDIS, 15782 Santiago de Compostela, Spain; irene.golan@usc.es (I.G.); lau.gz21@gmail.com (L.R.d.l.F.)

**Keywords:** brain tumor, malignant gliomas, glioblastoma, NK cells, immunotherapy

## Abstract

Glioblastoma (GB) is the most aggressive and most common malignant primary brain tumor diagnosed in adults. GB shows a poor prognosis and, unfortunately, current therapies are unable to improve its clinical outcome, imposing the need for innovative therapeutic approaches. The main reason for the poor prognosis is the great cell heterogeneity of the tumor mass and its high capacity for invading healthy tissues. Moreover, the glioblastoma microenvironment is capable of suppressing the action of the immune system through several mechanisms such as recruitment of cell modulators. Development of new therapies that avoid this immune evasion could improve the response to the current treatments for this pathology. Natural Killer (NK) cells are cellular components of the immune system more difficult to deceive by tumor cells and with greater cytotoxic activity. Their use in immunotherapy gains strength because they are a less toxic alternative to existing therapy, but the current research focuses on mimicking the NK attack strategy. Here, we summarize the most recent studies regarding molecular mechanisms involved in the GB and immune cells interaction and highlight the relevance of NK cells in the new therapeutic challenges.

## 1. Introduction

For many years, brain tumors were mainly classified based on their histopathological features and associated with possible cells of origin and level of differentiation. However, during the last decades, an important amount of data about the genetic basis of this type of tumors has been generated, providing a better understanding of key molecular pathways involved in their pathogenesis. This has contributed not only to a new World Health Organization Classification of Tumors of the Central Nervous System [1], but also a way for implementing better and more appropriate therapeutic approaches. Malignant brain tumors, and namely glioblastoma (GB), despite having rare occurrence in adults, are huge burdens for patients and families due to poor patient survival compared to other cancers. Notwithstanding efforts made to develop new therapies for GB, none has substantially improved survival. Lately, immunotherapy appears as a promising therapeutic approach, and among the different types, Natural Killer (NK) cells may become an important tool for GB immunotherapy. Clearly, the relationship between GB microenvironment and immune escape and the role of NK cells in the gliomagenesis process has resulted in NK cell-based immunotherapy becoming an attractive promise for GB treatment.

## 2. Glioblastoma

The most common primary brain tumors of the Central Nervous System (CNS) are gliomas, with GB being the most aggressive one [1]. Conventional treatment of this kind of tumors combines several approaches such as surgery, radiotherapy, chemotherapy with Temozolomide (TMZ) [2]. However, the prognosis is still unfavorable; only 5% of patients survive more than 5 years post-diagnosis [3]. According to the WHO Classification of Tumors of the CNS, glioblastoma is a diffuse, grade IV glioma of the astrocytic lineage. Histological studies of this kind of tumors show an extreme cell heterogeneity, which is mainly characterized by cellular pleomorphism, diffuse growth patterns and variation of the mitotic activity [4]. Moreover, its high invasiveness allows the tumor infiltration to healthy tissues and the generation of a large network of vessels that promote the proliferation of the tumor mass [5]. Although the immune system is able to detect and eliminate cancer cells, the microenvironment of the glioblastoma has the ability to suppress this response through diverse mechanisms such as the secretion of a large number of substances that interact with immune cells blocking their action [6].

## 3. Mechanisms of Immunosuppression

The brain was classically considered an immune-privileged organ because the restriction of immune cells traffic into the CNS. The blood-brain barrier (BBB) and the cerebrospinal fluid (CSF) are responsible for controlling the entry of immune cells into the brain. In physiological conditions, the migration of this kind of cells into the CNS is limited. Alternative forms of access for immune cells into the brain are the choroid plexus, where they entry directly to the CSF space, and through structures called circumventricular organs (CVOs), which have fenestrated capillaries without endothelial BBB and they are strategically localized at the midline of the ventricular system [7,8]. In pathological states, such as malignant brain tumors, BBB can be disrupted, increasing the permeability of immune cells into the damaged area [9].

The immune system is designed to protect the organism from infections or tissue damage. It is composed of several cell types that have different functions to fight against cancer cells and eliminate them. For instance, cytotoxic T lymphocytes (CTLs) can produce the lysis of immunogenic tumor cells by means of the recognition of antigenic peptides on their surface. This recognition is possible because of the interaction of the T-Cell receptors (TCR) with the major histocompatibility complex (MHC) [10]. Although one escape mechanism carried out by other kinds of cancer cells is the downregulation of the MHC presence [11], GB cells express high levels of MHC class I molecules. In this kind of malignancies, the tumor microenvironment is the most responsible for the local immunosuppression.

In a tumorigenic environment, the function of the immune system is not only limited to defense, but it can contribute to the development of the tumor. Acute inflammation can recognize tumor antigens and activate their effector functions to eradicate tumor cells. However, chronic inflammation has a pro-tumor effect, contributes to DNA damage, activates angiogenesis, promotes extracellular matrix (ECM) remodeling and inactivates the antitumor adaptive immune response [12].

**Figure 1:**
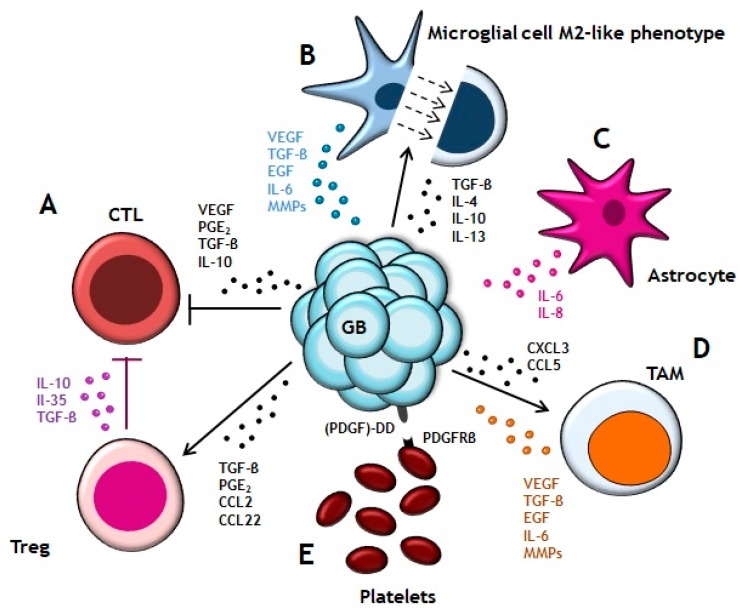
Glioblastoma (GB) microenvironment. (**A**) GB cells secret TGF-β, PGE_2_, CCL2 and CCL22 for recruiting Treg cells which contribute to the tumor progression by blocking CTLs’ cytotoxic activity. Additionally, GB cells also produce VEGF, PGE_2_, TGF-β and IL-10 for suppressing CTLs′ response and proliferation. (**B**) Liberation of TGF-β, IL-4, IL-10 and IL-13 promote the microglial cell transition to macrophage M2-like phenotype. In exchange, microglial cells secrete VEGF, TGF-β, EGF, IL-6 and MMPs to stimulate the tumor growth. (**C**) Astrocytes release IL-8 and IL-6 to stimulate VEGF expression in GB cells. (**D**) TAMs are recruited by gradient of chemokines (CXCL3 and CCL5). They participate in the angiogenesis and tumor expansion by producing VEGF, TGF-β, EGF, IL-6 and MMPs. (**E**) Interaction of (PDGF)-DD ligand, expressed in GB cells, with PDGFRβ receptor of platelets surface promotes tumor proliferation.

The GB microenvironment (Figure 1) is not formed only by tumor cells but it has the active participation of a wide variety of cellular components, including their interaction with the tumor mass. It is formed by the CNS cells, such as neurons and glial cells (astrocytes, oligodendrocytes and microglia); innate and adaptive immune system cells, such as monocytes and macrophages (tumor associates macrophages; TAMs), mast cells, neutrophils and T cells; endothelial cells and blood vessel; and ECM components. The tumor cells are able to manipulate their microenvironment and get that neighboring healthy cells cooperate in their development, maintenance and expansion [13]. GB cells secrete soluble factors, such as transforming growth factor beta (TGF-β), prostaglandin E2 (PGE2), CCL2 or CCL22 which recruit T regulatory lymphocytes (Tregs) to the tumor microenvironment [14,15,16]. Tregs contribute to pathogenesis and tumor progression by the production of interleukins (IL-10, IL-35) and TGF-β which block CTLs response [17]. In addition, vascular endothelial growth factor (VEGF), PGE2, TGF-β and IL-10 secreted by GB cells also suppress CTLs activity and proliferation [18]. There are several factors involved in microglial/astrocytic –GB cells crosstalk [19]. GB cells release TGF-β, IL-10, IL-4 and IL-13 that promote the microglial cells to acquire a phenotype similar to M2 macrophages. M2-like phenotype has been associated with high aggressiveness and poor prognosis in GB patients [20]. In turn, microglial cells secrete, among other factors, TGB-β for stimulating the tumor mass expansion [21]. Astrocytes also contribute to tumor migration and invasion by release IL-8 or IL-6 which promotes expression of VEGF in tumor cells [22]. Recruitment of TAMs to GB lesion is based on the gradient of chemokines and cytokines, such as CX3CL1 or CCL5, which are released by tumor cells [23,24]. TAMs collaborate in angiogenesis through secretion of different substances among which stand out VEGF, epidermal growth factor (EGF), TGF-β, IL6 and matrix metalloproteinases (MMPs) that remodel the ECM in order to facilitate tumor growth [6]. Although, it was reported that, both in glioma cell lines and primary GB tumor tissues, express platelet-derived growth factor (PDGF)-DD protein, a ligand able to activate mitogenic pathways in these cells through platelet-derived growth factor receptor beta (PDGFR this growth factor alerts tumor-infiltrating innate immune cells through engagement of NKp44, present in GB infiltrating NK cells. This mechanism may provide a novel therapeutic approach based on blocking PDGFR and favoring PDGF-DD-mediated stimulation of NKp44^+^ NK cells [25].

## 4. The Role of NK Cells on GB

NK cells are granular lymphocytes of the innate immune system. They secrete cytokines and chemokines that participate in the immune response. Their peculiarity is that they can recognize target cells by the lack of MHC molecules on the surface, and kill them without previous activation. The death of infected or transformed cells is mediated by perforin/granzyme pathway or death receptor-related pathways [26]

The cytotoxicity process of NK cells initiates with their surveillance feature. They have short and exploratory interactions with many different types of cells in the organism. When NK cells interact with their target cell, they set up an intercellular connection by means of membrane nanotubes. These structures are heterogeneous and can transmit the signal over a large distance and supply lytic granules [27]. The recognition of many ligands of host and pathogens by NK cells is mediated by different activating receptors. They are classified on the basis of their intracellular signaling motif: immunoreceptor tyrosine-based activation motif (ITAM) such as NKp30, NKp44, NKp46 and CD16; immunoreceptor tyrosine-based switch motif (ITSM) such as 2B4, NTB-A and CRACC; immunoreceptor tyrosine tail (ITT)-like motif such as NKG2D and DNAM-1; and finally, hem-ITAM in their cytoplasmic tail such as NKp65 and NKp80. The activation of this receptors stimulates the effector function of NK cells. However, for the complete activation of resting NK cell, is necessary the interaction of, at least, two different activating receptors with their respective ligands. The activation can be enhanced with the participation of different cytokines such as IL-2, IL-12, IL-15, IL-18 and IL-21 [28]. After the recognition of the target cell, NK cell generates a lytic synapse that involves two main domains: a peripheral domain, formed by a ring-type structure which represents the point of contact between cells and that contains adhesion molecules such as the integrin LFA-1; and the central domain, which is the focal point of the exocytosis [29].

The effector stage is characterized by the actin polymerization and cytoskeletal reorganization at the lytic synapsis. In this stage, lytic granules move quickly through microtubules to converge on microtubule-organizing center (MTOC). This process represents the last moment in which NK cells are susceptible to inhibitory signaling before getting engaged with cytotoxicity. The next step is the polarization of MTOC. At this point, associated lytic granules move towards synapsis and NK cells proceed to degranulation [29,30]. The reorientation of the cell organelles, such as the placement of Golgi apparatus together with the microtubules and near the synapse, helps to secrete the granules′ contents to the target cell [31]. Mitochondrial polarization is also important because it promotes the adequate influx of Ca^2+^, which is required for a correct signaling and exocytosis, and it is the main energy sources necessary to enhance de synaptic function [30]. The docking of lytic granules with the plasmatic membrane happens before the fusion between the two membranes. This process can come about in two ways: (1) a complete fusion with the release of all the content of granules, and (2) an incomplete fusion with the formation of a transitory pore in the plasmatic membrane and a partial liberation of the content [32].

The last stage is the termination, perforin released by NK cells induces a membrane flipping on the target cell, which allows phosphatidylserine to be exposed on the extracellular surface of the cell. This is the signal for NK cells to finish the response. The detachment takes place, and the cytotoxicity arrives at its end. Then, NK cells can initiate a new phase of recognition or just remain in a resting state [30].

The granule-based killing pathway consists of the discharging of perforin by NK cells. Perforin is a pore-forming protein that pierces the plasmatic membrane on the target cell and allows the input of serine proteases, called granzymes, to the cytosol. If target cell could not neutralize this harmful effect on its membrane, it will activate its own cell death by necrosis. Otherwise, if the target cell can restore the integrity of its membrane, granzyme would induce cell death through caspase cascade activation and subsequent apoptosis [33]. 

There are two cell death mechanisms, independent of degranulation, by which NK cells mediate cytotoxicity: Fas-ligand (FasL) and the tumor necrosis factor-related apoptosis-induced ligand (TRAIL). Both of them are ligands on the NK cell surface and interact with their correspondent receptor localized on the plasmatic membrane of the target cells. FasL interacts with Fas and TRAIL has several receptors, but only two of them have the death domain motif in their intracellular region. This domain is required for the apoptosis signaling. The ligand-receptor complex forms the death-inducing signaling complex (DISC) that recruit the Fas-associated death receptor (FADD) and caspases-8 and -10. The proximity between these factors favors the caspases autoproteolytic process and release of the active proteases. In type I cells, active caspase-8 is sufficient to directly active other members of caspases family that, finally, activate the apoptosis phase. In type II cells, mitochondrial translocation of caspase-8 cleaves Bid, a proapoptotic Bcl2 family member, whose mitochondrial translocation allows its interaction with Bax and Bak and the subsequent release of cytochrome-c and SMAC/DIABLO to the cytosol. In the cytosol, SMAC/DIABLO binds to inhibitors of apoptosis proteins, promoting the apoptosis activation, and cytochrome-c binds to APAF-1 to form the apoptosome. The apoptosome activates caspase-9, which in turn activates caspase-3, -6 and -7. Caspase-3 process, death substrates and caspase-8 outside the DISC, completing, a positive feedback loop [34,35].

It is important to mention the antibody-dependent cellular cytotoxicity (ADCC) because this mechanism is the main mechanism of some antibodies used in NK-mediated tumor therapies, such as cetuximab [36]. Fcγ Receptors (FcγRs) recognizes part of Fc from IgG antibody with different affinity. On NK CD56^dim^ CD16^+^ cells, FcγRIIIa (type III receptor for IgG, CD16) is highly expressed, and this receptor is responsible for ADCC. NK cells are considered the main mediator of the ADCC because they not co-express the inhibitory FcγRIIb. However, the co-expression of other activating receptors has a synergic effect and increases the NK activation. The complex formed by IgG1 or IgG3 with FcγRIIIa induces a potent activating signal that is higher than inhibiting signals, and mobilizes a cytotoxic response [37,38]. NK cells are defined by the expression of CD56 (neural cell adhesion molecule) and the absence of the T-cell co-receptor CD3. Regarding the surface expression of the CD56 protein, there are two sets of NK cells: the CD56^bright^ subset, that comprises 10% of the circulating NK cells, is more proliferative but shows lower cytotoxicity toward cancer cells; and the CD56^dim^ subset, that comprises the majority of circulating NK cells (90%) and has a higher capacity to recognize and kill target cells [39,40]. Although CD56^dim^ NK cells are predominant in the blood, CD56^bright^ are the most abundant NK subset in the human body because of their concentration in lymphoid and non-lymphoid tissues. CD56^bright^ NK cells have been identified in lymphoid tissues, liver and uterus. All of them have an altered phenotype regarding their CD56^bright^ circulating counterparts and the acquisition of this different phenotype is related with the tissue-specific function. Based on the expression of CD69, chemokine receptors and adhesion proteins, tissue-resident CD56^bright^ NK cells can be distinguished from circulating CD56^bright^ NK cells [41]. We have to mention that all CD56^dim^ peripheral blood NK cells have also the CD16 receptor on their surface, but after their activation CD16 expression is decreased, and interferon-γ (IFN-γ) production is increased [42]. It has been observed that the increment of IFN-γ produced by NK cells is associated with improved survival in GB patients [43]. NK cells express both activating and inhibitory receptors on their surface. The interaction between one of these receptors with its corresponding ligand on the target cell surface will determine the action of these lymphocytes [44].

A recent classification of NK cells emerges from a cytomegalovirus (CMV) infection context or vaccines response. Here we have the canonical NK cells which are the typical NK cells characterized by being immunoregulators and they have the capacity of maintaining the immune homeostasis. This type of cells triggers the death signal through their contact with the target cells. This effect is increased with the stimulation of IL-12 and IL-18 or engagement of the low-affinity FcRCD16. On the other hand, we have the adaptive NK cells, the “new” population which carries out the immunosurveillance of infected cells and have the ability to survive and proliferate in an inflammatory context. This population is characterized by having a long-term immunological memory, low response to IL-12 and IL-18, degranulation capacity and cytokines production in response to FcRCD16 engagement. Nonetheless, the most particular features are their self-renewal capacity and long half-life. The existence of these two different NK populations is important to be taken into account in NK-based therapy. The therapy with canonical NK cells is based on a short anti-cancer strategy due to the short half-life of these cells. For this reason, it is required to prolong the persistence of adoptively transferred NK cell therapy. The use of the adaptive population in the clinic is an improvement because, with their self-renewal capacity and long half-life, it is possible to avoid the disadvantages mentioned previously and have NK cells ready to use in case of patient relapse [45,46].

NK cells are the least abundant population of all immune cells infiltrate in the glioma. They represent 2.11% of the total and the most abundant phenotype is CD56^dim^CD16^neg^ [47]. This phenotype was previously reported to show considerable activation in other tumors [48]. This means that, despite the quantity of NK cells in GB microenvironment is low, they have cytotoxic activity. Therefore, development of new tools that allow an increase in the number of NK cells in GB tumors could improve their oncolytic function. For example, an analysis of the GB cell surface molecules revealed that they present high levels of MHC class I molecules and human leukocyte antigen (HLA)-A, HLA-B and HLA-C ligands. All of them interact with inhibitory receptors, like the killer immunoglobulin-like receptors (KIRs), and inhibit NK cells function. If the interaction among MHC molecules and KIRs is blocked, it would allow NK cells to attack GB. In this case, the number of NK cells infiltrating the GB microenvironment would be increased and they could carry out their function.

KIR family of NK receptors is composed of 12 members that can be divided into two functionally different subsets: six members are activating receptors and six members are inhibitory receptors. The difference between these two receptor subsets resides in the transmembrane and intracellular domain. The extracellular domain is the same for all of them. These receptors function as monomers that can contain two or three immunoglobulin-like domains (KIR2D or KIR3D). Regarding its intracellular region, this can be long (L; KIR2DL or KIR3DL) and generate an inhibitory signal through the immunoreceptor tyrosine-based inhibition motifs (ITIMs), or short (S; KIR2DS or KIR3DS) and develop an activating signal through its association with adaptor proteins that contain ITAMs [49]. The inhibitory signal blocks activating signals that come from activating receptors, but this block is only local and does not affect the NK cell’s stimulation. Inhibitory signaling mediated by KIR initiates with the recruitment of the tyrosine phosphatase Src homology region 2 domain-containing phosphatase-1 (SHP-1) on phosphorylated ITIM domains of the receptor. SHP-1 dephosphorylates and inactivates the guanine exchange factor Vav1. Moreover, the inhibitory receptor phosphorylates the adaptor molecule Crk through the tyrosine phosphatase c-Abl, and phospho-Crk dissociates from the cytoskeletal signaling. Both Viv1 and Crk are needed in activating signals [50].

GB cells express molecules on their surface, such as MHC class I polypeptide-related sequence A (MICA), that interact with activating receptors like NKG2D+ present on NK cells membrane. This interaction has a great impact on GB lysis. It was seen that NK cells attenuate their activity against GB when NKG2D+ receptor is blocked [51]. Additionally, CD155 and CD112 overexpression in tumor cells may induce NK cytotoxicity by their interaction with TIGIT and CD96 receptors [52].

It is important to consider that some frequent mutations in glioblastoma, such as the upregulation of growth factor signaling pathways and/or the loss of cell cycle regulators, allow glioblastoma cells to escape from immune surveillance through the resistance to NK-derived cytotoxicity [53]. By contrast, and not less relevant, is the identification of tumor cells susceptible to the NK-mediated cytotoxicity. It has been reported that undifferentiated GB cells, and precisely GB cells with stem cell properties, are more susceptible to NK cell lysis through upregulation of activating and downregulation of inhibitory ligand for NK cell receptors [54,55].

## 5. Therapeutic Approaches in GB with NK Cells

GB therapy has been improved since the first resection surgery. Currently, the gold standard treatment is the surgery with radiotherapy alone or combined with chemotherapy. However, new strategies are being investigated to overcome this cancer. Some of these trials are based on immunotherapy and the use of NK cells (Table 1).

GB immunotherapy is based on the use of different immune cells, such as dendritic cells, cytotoxic T lymphocytes, and NK cells. These last ones were those that showed the strongest cytotoxic activity against malignant tumor cells [78], and they are less toxic alternative than chemotherapy and radiotherapy [76].

When we refer to cell therapy, we can distinguish two strategies: autologous and allogeneic therapy. Cells and tissues are collected from the patient, cultivated and expanded ex vivo and reintroduced back into the donor. This strategy has some advantages such as the low risk from immune system reaction, biocompatibility and no risk of disease transmission associated with the transplant. However, there are some disadvantages such as the requirement of sophisticated set-up, well-trained personnel and the high cost of the treatment. It is also important to take into account that the cell or tissue processing and preparation takes a long time and, as a consequence from extraction to their use, there is a lapse of time that delays the treatment. Another problem is the inhibitory response that arises from the recognition of own MHC molecules by NK cells [79,80]. There are few clinical trials based on autologous NK cells because of their complicated selection and expansion from patients’ peripheral blood mononuclear cells (PBMCs). One of them has been performed by Ishikawa et al. and they achieved a reduction of the tumor volume after the treatment. These researchers have proposed that this response could be increased if the treatment is combined with an appropriate IL-2 dose or radiation therapy [65]. Another type of autologous therapy is the utilization of lymphokine-activated killer cells (LAK). LAK cells derive from lymphocytes which have been cultured with IL-2. GB in vitro assays have shown that LAK cells can destroy autologous GB cells, but not kill autologous lymphocytes. This idea has evolved until it reached the phase II trial, in which LAK cells and IL-2 was administered into the CNS. The last results show a higher survival rate in patients with recurrent glioma [67,81].

**Figure 2:**
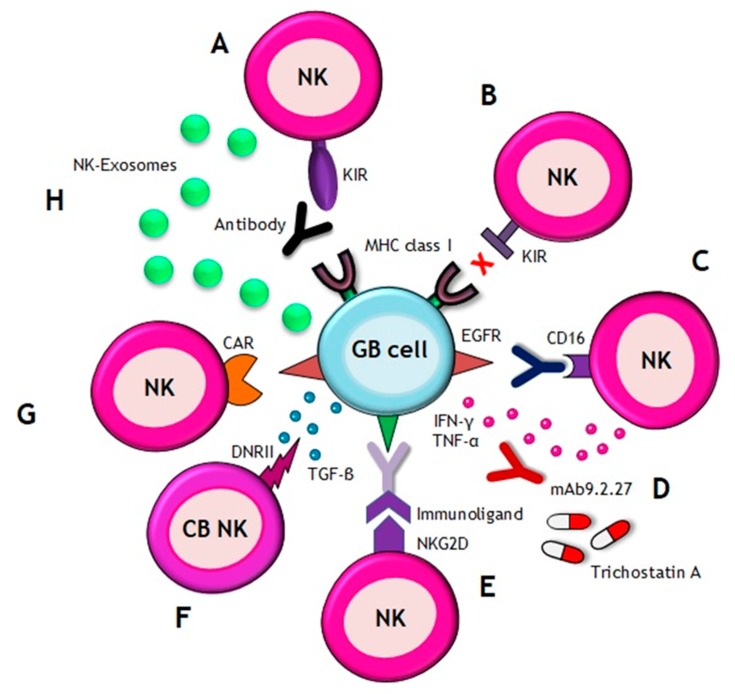
Natural Killer (NK) cell-based immunotherapy. There are different approaches of immunotherapy in GB, including (**A**) the use of the antibodies which inhibit the interaction between KIRs on NK cells and MHC class I on GB cells, (**B**) transference of allogenic NK cells that express different KIRs and, thus, they are notable to recognize MHC class I on GB cells, (**C**) the use of antibodies against EGFR, which are recognized by CD16 receptor of NK cells, (**D**) combination of NK cells with the following: drugs, such as trichostatin A, for sensitizing GB cells to the NK attack; or with mAb9.2.27 antibody for inhibiting angiogenesis by secretion of IFN-γ and TNF-α, (**E**) the use of immunoligands conjugated to a NKG2D receptor on NK cells, which have the ability to recognize tumor-specific antigens, (**F**) the use of cord blood (CB) NK cells expressing a dominant negative TGF-β receptor II (DNRII) which allow NK cells being activated even in presence of TGF-β, (**G**) the use of NK cell line carrying a CAR targeting EGFR variant III (expressed in tumors) for inducing apoptosis and (**H**) the utilization of NKs-derived exosomes which promote apoptosis.

It is well known that the graft of foreign tissue develops rejection, but in the cell-based therapy field, there have been cells identified with the ability to evade or suppress the immune system [82]. This is the basis of the use of allogeneic cell therapy, very important in NK cell context. Previously, we have mentioned the ability of KIR receptors to recognize MHC class I molecules on tumor cells, promoting the inhibitory signal that allows tumor cell survival. This circumstance has promoted the use of allogeneic NK cells, which come from a totally unrelated donor. KIR receptors from the donor are unable of recognize MHC class I molecules of the acceptor patient (Figure 2B). This approach results in a lack of recognition of tumor MHC molecules by the KIRs present on the allogeneic NKs and, as consequent, there is an absence of inhibitory signals allowing NK cells activation [39,64]. This strategy has been tested in patients with renal cell cancer and melanoma [68]. Their results show a successful and safe expansion of NK cells in advanced cancer patients. Moreover, adoptive transfer of allogeneic NK allows for a more flexible selection of cells by expanding and cryopreserving them for an immediate administration, without waiting for the expansion time ex vivo [83].

An alternative could be the use of specific antibodies against KIRs for blocking their union with the MHC class I ligands, avoiding the inhibitory response produced by GB cells (Figure 2A). This therapeutic approach has been already investigated for testing their safety in multiple myeloma patients [70]. This study describes the efficiency of an anti-KIR antibody called IPH2101 for enhancing NK cell functions against tumor cells.

On the other hand, a subset of KIR triggers activating signals. These receptors can recognize the same ligands than inhibitory receptors but with a lower affinity. However, KIR2DS2 has been identified as potent activating receptor against GB cells, and its effect is independent of the rest of inhibitory or activating receptors present on the NK cell surface. KIR2DS2 has shown to induce a dominant signal able to mask the effect of inhibitory receptor associated at its ligand. After seeing the results of KIR2DS2 on the decrease of cell proliferation and angiogenesis, and on the increase of apoptosis, it has been proposed as an important clinical criterion in the NK cell donor selection for so improving the effectiveness of cell-based anticancer therapy [51].

One strategy, already tested in animals, is the combination of NKs treatment with the monoclonal antibody mAb9.2.27 [72]. This antibody inhibits the angiogenesis and together, with NK cells, secreting IFN-γ and TNFα. Tumor proliferation was reduced, prolonging survival of rats with GB (Figure 2D).

Focusing in CD16 receptor on NK cells which is able to recognize the Fc part of antibodies [84], it would be possible to induce the apoptosis of GB cells by means of antibody-dependent cellular cytotoxicity [38]. For example, cetuximab, used for inhibiting epidermal growth factor receptor (EGFR) on cancer cells, can be recognized by NK cells through CD16 receptors and produce apoptosis (Figure 2C). The use of NK cells in anti-EGFR therapies overcomes the limitations of the last ones. It has shown that NK cells increase the efficiency of therapies based on anti-EGFR antibodies in a RAS^mut^, BRAF^mut^ or EGFR^low/-^ colorectal cancer context. NK cells are capable to lyse primary colon cancer cells independently of the RAS, BRAF and EGFR status, but this effect is enhanced in combination with cetuximab in EGFR-positive cells through the activation of ADCC [36]. Another interesting approach is the use of immunoligands. These kinds of fusion proteins, which have the ability to recognize tumor-specific antigen, are conjugated to an activating receptor on NK cells, like NKG2D (Figure 2E). In prostate carcinoma, they have shown an antitumoral effect both in vitro by enhancing cell lysis, and in vivo by inhibiting tumor growth significantly. It has been seen that immunoligands recruitment, cross-link and activate NK cells to tumor cells to promote lysis of cancer cells independently of MHC class I molecules or NK ligands [69].

Due to immune-evasion strategies of cancer cells, some approaches are focused on the use of drugs in combination with NK cells for sensitizing the tumor. A histone deacetylase inhibitor (HDACi), call trichostatin A, can induce MICA expression in GB cells allowing to increase NK cell cytotoxicity (Figure 2D). In vivo experiments show that trichostatin A improves the recognition of GB and promote NK cell-mediated lysis [71].

The most recent findings concern to GB immunotherapy include the use of cord blood NK cells [73], exosomes derived from NKs [76] and a novel NK cell line carrying a chimeric antigen receptor (CAR) targeting EGFR variant III [77]. Cord blood (CB) NK cells seems to be a promising cellular treatment. Though, due to immune-suppressive microenvironment of GB, it is necessary to modify CB-derived NKs for counteracting the effects produced by the cytokines secreted by GB cells. TGB-β inhibits expression of activating receptors such as NKG2D, thus, a dominant negative TGF-β receptor II (DNRII) was engrafted on CB-derived NKs (Figure 2F). DNRII CB-derived NKs have the ability to be activated even in presence of TGF-β [73].

NK-derived exosomes (NK-Exo) have been used to treat incurable cancers such as melanoma [85]. Recently, it has been investigated their antitumor effect against GB (Figure 2H). NK-Exo promote apoptosis of GB cells in vitro and inhibit in vivo tumor growth in mice [76]. Furthermore, it has been studied the use of exosomes like delivery drug systems in CNS diseases. BBB inhibits 98% of all methods that are potential treatments for the cure of CNS diseases. Exoxome-based therapy offers one relevant advantage, which is that these nanovesicles have the appropriate size -about 100 nm- to cross the BBB and release their content towards the target cells. So, NK cells derived exosomes can be loaded with drugs enhancing their antitumoral effects and tumor specificity, or simply they can act as carriers of anticancer agents across the BBB to the affected brain region [76].

Finally, a NK cell line carrying a CAR targeting EGFR variant III, expressed in tumors, was established for inducing apoptosis in glioblastoma cells (Figure 2G). Results were evaluated in vitro and they indicate this cell line as an effective treatment option for patients with GB [77]. A new strategy in this field is the development of CAR-engineered variants on the NK-92 cell line. One of them is ErbB2-specific NK-92/5.28.z cells (HER2.taNK), at present in phase I of clinical trials. It has been demonstrated that this ErbB2-NK cell line can recognize both trastuzumab-resistant ErbB2-positive breast carcinoma cells and trastuzumab-sensitive ErbB2-positive breast carcinoma cells, including those cells with low target antigen expression on their surface. These cells exhibit great efficiency through ADCC and serial killing, and their cytotoxicity is not affected by hypoxia, normally present in GB tumors. Neurospheres are sensitive to this cytotoxicity and all tumor cells are eliminated. Orthotopic xenografts model in NOD-SCID IL2Rγ null mice and stereotactic injection of HER2.taNK in tumor area have displayed inhibition of the tumor progression. These in vitro and in vivo assays have shown a potent and selective antitumoral activity of ErB2-specific NK-92/5.28.z, not only in a direct manner but also through the production of a large amount of pro-inflammatory cytokines and the induction of a protective endogenous antitumoral immunity after the treatment [74,75].

## 6. Conclusions

In summary, immunotherapy with NK cells seems to be a promising strategy for treating GB patients. Furthermore, the use of techniques that increase direct cell-to-cell contact between GB cells and NK cells could potentiate the antitumor effect. However, a deeper investigation of the therapeutic role of NK cells in the context of GB is necessary.

## Figures and Tables

**Table 1:** GB therapy timeline. Evolution of NK-based immunotherapy.

Year	Events	Ref.
1884	First recognized surgery for primary brain tumor resection	[56]
1940s	Implementation of radiotherapy in the treatment of brain tumors	[57]
1950s	First chemotherapy session	[58]
1970s	Incorporation of computed tomography (CT) into radiotherapy planning	[57]
	Synthesis of TMZ	[2,58]
	Emergence of immunotherapy against gliomas concept	[59,60]
1980s	Incorporation of magnetic resonance imaging (MRI)	[57]
1993	It is discovered that combination of chemotherapy with radiotherapy increases patients survival	[61]
Nowadays	Gold standard therapy for GB patients-surgery in combination with radiotherapy and/or chemotherapy	[61]
	FDA has approved TMZ like chemotherapeutic agent for GB patients	[2]
	Development of new strategies:	
	Tumor treating fields (TTF): application of low intensity electric fields on brain tumors. FDA has approved the use of TTF in early diagnosis and recurrent GB patients	[62,63]
	New disvoveries in the use of nanosystems against GB	
	Advances in NK cell-based immunotherapy:	
2002	The use of allogeneic NK cells expressing KIR receptors which are not able to recognize the patient’s MHC-class I molecules	[39,64]
2004	Autologous NK cell therapy for recurrent GB	[65]
2005	The use of cord blood as a source of NK cells	[66]
2006	Phase II trial: intralesional autologous LAK cells therapy in recurrent GB patients. This study has been completed in 2013	[67]
2008	Phase I trial: allogeneic NK-92 cell line therapy in patients with advanced renal cell cancer or melanoma	[68]
2011	Induction of NK cell cytotoxicity using a immunoligand conjugated to a NKG2D receptor that recognizes tumor specific antigens. This approach has been tested in prostate carcinoma cell lines	[69]
2012	Phase I trial: therapy with an anti-KIR antibody in multiple myeloma patients. This antibody blocks the binding of KIR with MHC-class I molecules and avoids the inhibitory response of NK cells	[70]
2013	Combination of NK cells with Trichostatin A induces MICA expression on GB cells allowing to increase NK cytotoxicity	[71]
2014	Combination of NKs with mAb9.2.27 antibody changes the GB anti-Inflammatory microenvironment into a pro-inflammatory one	[72]
	Identification of KIR2DS2 as a potent activating receptor on alloreactive NK cells which decreases cell proliferation and angiogenesis GB tumors	[51]
2016	Apoptosis mediated by CD16 receptors after the recognition of antibodies, such as cetuximab, which are blocking EGFR on tumor cells. This approach has been tested in colorectal cancer cell lines	[36]
2017	Cord blood-derived NK cells expressing a dominant negative TGF-β receptor II (DNRII) recognize and kill GB tumor cells even in the presence of TGF-β	[73]
	Phase I trial: intracranial injection of ErbB2-specific NK-92/5.28.z (HER2.taNK) cells in patients with recurrent HER2-positive GB	[74,75]
2018	Study of NK cells-derived exosomes as potential immunotherapeutic agents for cancer treatment	[76]
	Establishment of a chimeric antigen receptor (CAR) on NK cell line which recognizes EGFRvIII and promotes apoptosis in GB cells	[77]
Preclinical studies; Clinical studies		

## Abbreviations

BBB	Blood-brain barrier
CAR	Chimeric antigen receptor
CB	Cord blood
CSF	Cerebrospinal fluid
CNS	Central Nervous System
CTL	Cytotoxic T lymphocyte
CVO	Circumventricular organ
ECM	Extracellular matrix
EGF	Epidermal growth factor
EGFR	Epidermal growth factor receptor
DISC	Death-inducing signaling complex
DNRII	Dominant negative TGF-β receptor II
FADD	Fas-associated death receptor
FasL	Fas-ligand
GB	glioblastoma
HDACi	Histone deacetylase inhibitor
HLA	Human leukocyte antigen
IL	Interleukin
ITAM	Immunoreceptor tyrosine-based activation motif
ITIM	Immunoreceptor tyrosine-based inhibition motif
ITSM	Immunoreceptor tyrosine-based switch motif
ITT	Immunoreceptor tyrosine tail
KIR	Killer immunoglobulin-like receptor
LAK	Lymphokine-activated killer
MHC	Major histocompatibility complex
MICA	MHC class I polypeptide-related sequence A
MMP	matrix metalloproteinase
MTOC	Microtubule-organizing center
NK	Natural Killer
PBMC	Peripheral blood mononuclear cell
PDGF	Platelet-derived growth factor
PDGFR	Platelet-derived growth factor receptor beta
PGE2	Prostaglandin E2
SHP-1	Src homology region 2 domain-containing phosphatase-1
TAM	Tumor-associated macrophage
TCR	T-Cell receptor
TGF-β	Transforming Growth Factor beta
TMZ	Temozolomide
TRAIL	Tumor necrosis factor-related apoptosis-induced ligand
Treg	Tregulatory lymphocyte
VEGF	Vascular endothelial growth factor
